# Individual Differences in the “Cognitive–Adaptive Gap” Among Children with Autism Spectrum Disorder: A Latent Profile Analysis of the Moderating Role of Family Environment

**DOI:** 10.3390/jintelligence14060103

**Published:** 2026-06-09

**Authors:** Ning Shao, Lingling Wu, Wenhao Li, Chao Song, Wenyuan Jin, Lifei Hu, Xiuchun Zhang, Zhiwei Zhu

**Affiliations:** Department of Developmental-Behavioral Pediatrics, The Children’s Hospital, Zhejiang University School of Medicine, National Clinical Research Center for Children’s and Adolescents’ Health and Diseases, Hangzhou 310052, China; shaoning8023@163.com (N.S.);

**Keywords:** human intelligence, adaptive behavior, WISC-IV, autism spectrum disorder (ASD), Latent Profile Analysis (LPA)

## Abstract

This study investigates the “competence–performance gap” between cognitive ability (measured by the WISC-IV) and actual adaptive performance (measured by the ABAS-II) in children with autism spectrum disorder (ASD), and examines the moderating role of family environment, specifically parental education levels. We applied Latent Profile Analysis (LPA) to cross-sectional data from 3246 children with ASD (aged 6–16 years). The analysis identified three distinct cognitive–adaptive subgroups: the Balanced High-Functioning group (33%), the Classic Mismatch group (44%), and the Cognitively Vulnerable group (23%). Notably, the Classic Mismatch group was characterized by adaptive performance that significantly trailed cognitive potential. Multinomial logistic regression revealed that maternal education—but not paternal education—significantly predicted a child’s likelihood of being in the “Balanced High-Functioning” group. This moderating effect was especially pronounced during the school-age years. These findings highlight the critical role of environmental factors in the translation of intellectual potential into practical social adaptive functioning, providing theoretical support for targeted family-based interventions.

## 1. Introduction

Human intelligence encompasses more than just the cognitive potential measured by standardized tests (i.e., “competence”). Crucially, it involves “performance”—an individual’s ability to translate this potential into adaptive behaviors for solving real-world problems. This competence–performance gap is a central theme in intelligence research, reflecting individual differences in the efficiency of converting cognitive potential into daily functional outcomes (Sternberg et al., 2021). Understanding this gap is not only key to deciphering the mechanisms of intellectual translation but also serves as a core indicator for assessing functional prognosis. Furthermore, such individual variations may hold greater theoretical significance than group-level mean differences (Radtke et al., 2025). Autism Spectrum Disorder (ASD) provides a unique window into the study of this competence–performance gap. ASD is primarily characterized by persistent deficits in social communication and the presence of restricted, repetitive patterns of behavior (APA, 2013). Recent monitoring data from the U.S. CDC indicates that the prevalence of ASD among 8-year-old children has reached 1 in 31 (Shaw et al., 2025). Given the clinical complexity of the autism spectrum, clinicians often use a multimodal approach (combining professional and objective behavioral observation, standardized neuropsychological assessment, and a comprehensive developmental history) to ensure the accuracy of ASD diagnosis. While previous studies have predominantly relied on Intelligence Quotient (IQ) as the primary metric for assessing cognitive ability in children (Audras-Torrent et al., 2021; Giofre et al., 2024), our team’s preliminary research suggests that IQ alone does not fully predict social adaptive functioning in children with ASD (Jin et al., 2025; W. Li et al., 2024). This discrepancy is largely attributed to the interference of core ASD symptoms and deficits in real-world executive control, which often act as barriers to the functional application of cognitive potential (Kenworthy et al., 2010). Consequently, a significant “chasm” exists between an individual’s cognitive capacity and their daily adaptive performance (Alvares et al., 2020). Recent research has further conceptualized this phenomenon as the ‘Cognitive–Adaptive Gap’, highlighting that such discrepancies are prevalent among children with ASD and are significantly modulated by environmental and mediating factors (Wang et al., 2024). This suggests that a unitary IQ-based classification may mask diverse clinical needs. Latent Profile Analysis (LPA) offers a robust framework to integrate multi-dimensional developmental indicators, thereby identifying functional subgroups that IQ metrics alone cannot distinguish (Kaneko et al., 2023; S.-J. Liu et al., 2025; S. Liu et al., 2025a; Lubke & Muthen, 2005). Existing literature confirms that this gap exists widely. However, its heterogeneity across different cognitive phenotypes remains unclear. How environmental factors—such as the family ecosystem—modulate this process is also poorly understood. These gaps represent a critical, under-explored topic at the intersection of intelligence science and clinical psychology.

Within the field of intelligence research, Family Socioeconomic Status (SES) serves as a pivotal environmental moderator. Existing literature suggests that the real-world expression of intelligence is not solely determined by genetic or biological factors but is profoundly shaped by the family ecosystem (Gustavson et al., 2025). Specifically, parental educational background—a stable indicator of household SES—is regarded as a crucial external resource during the developmental trajectory of children with ASD (Cao et al., 2025). However, prior studies have frequently aggregated maternal and paternal education into a composite measure (Milgramm et al., 2022; Morsa et al., 2022), thereby overlooking the potential asymmetry in their respective effects and limiting our understanding of environmental moderating mechanisms. Mothers often bear a disproportionate share of responsibility in daily interventions for children with ASD. Therefore, it is crucial to examine parental education effects separately. This will help uncover how environmental factors moderate the competence–performance gap. Furthermore, cognitive and adaptive development in children with ASD is not static but evolves dynamically with age. As children transition into and through their school-age years, societal demands for independence and social complexity increase sharply (Mahendiran et al., 2019; C. Song et al., 2024). Regarding the developmental trajectory of ASD, two distinct perspectives have emerged. One posits that early intervention can effectively narrow developmental gaps (Klintwall et al., 2015). Conversely, the other suggests that as environmental demands intensify with advancing grade levels, the discrepancy in adaptive functioning may instead widen, leading to an increasingly pronounced lag in functional living skills (McQuaid et al., 2021). The consistency of this age-related evolution across diverse ASD phenotypes is unknown. This is especially pressing for individuals with severe intellectual impairment, where the gap may widen faster.

Building upon these considerations, the current study utilizes a large dataset of 3246 cases. We employ Latent Profile Analysis (LPA)—a person-centered methodological approach—to systematically identify heterogeneous phenotypes of cognitive and adaptive functioning within the ASD population. We aim to elucidate how the family ecosystem moderates the translation of intellectual potential into social adaptive functioning. Specifically, our research objectives are threefold: first, to identify the latent profiles of the cognitive–adaptive relationship and characterize their intellectual phenotypes, thereby demonstrating both the universality and specificity of the “competence–performance gap”; second, to examine the predictive role of parental education levels on children’s profile membership from a family-school ecological perspective, with a particular focus on the asymmetrical effects of maternal versus paternal education on the expression of intellectual potential; and third, to investigate the dynamic age-related patterns of the competence–performance gap across different profiles as children age, exploring the patterns by which intellectual translation efficiency evolves over time. This study deeply analyzes the ‘chasm’ between intellectual potential and real-world performance. In doing so, we aim to provide interdisciplinary evidence that can optimize contemporary intelligence assessment paradigms and inform differentiated support strategies.

## 2. Materials and Methods

### 2.1. Participants

This study retrospectively reviewed data from 3246 children diagnosed with ASD at the Children’s Hospital of Zhejiang University School of Medicine between January 2020 and December 2024. Inclusion criteria required participants to be 6–16 years old, have a DSM-5 ASD diagnosis independently confirmed by two developmental–behavioral pediatricians, and possess complete WISC-IV and ABAS-II assessment data. Exclusion criteria included comorbid emotional disorders, epilepsy, visual or auditory impairments, cerebral palsy, Down syndrome, or other identified genetic intellectual disabilities. All participants received a formal diagnosis of ASD from senior pediatric psychiatrists in specialized clinical settings, based on the DSM-5 criteria and comprehensive developmental histories. At the time of evaluation, approximately 60% of the participants were enrolled in community-based intervention programs, primarily focusing on Applied Behavior Analysis (ABA).

The final sample consisted of 2473 boys (76.2%) and 773 girls (23.8%), with a mean age of 8.03 years (SD = 1.05). Collected demographic information included the child’s sex and age, as well as parental education levels (categorized into five levels: primary school or below, junior high school, senior high school/vocational school, junior college/bachelor’s degree, and postgraduate degree or above).

### 2.2. Measures

#### 2.2.1. Wechsler Intelligence Scale for Children, Fourth Edition (WISC-IV)

The WISC-IV is a standardized instrument for assessing cognitive functioning in children aged 6–16 years. The Chinese version was standardized in 2007 (Zhang, 2009), and has been extensively validated for its robust reliability and validity among Chinese children (H. Chen et al., 2010). In this study, we utilized the standard scores (M = 100, SD = 15) for the Full-Scale Intelligence Quotient (FSIQ) and four primary indices: the Verbal Comprehension Index (VCI), Perceptual Reasoning Index (PRI), Working Memory Index (WMI), and Processing Speed Index (PSI). All assessments were administered by psychometrists who had undergone standardized training.

#### 2.2.2. Adaptive Behavior Assessment System, Second Edition (ABAS-II)

Daily adaptive functioning was evaluated using the ABAS-II (Y. Li & Qiu, 2016). This study utilized the General Adaptive Composite (GAC) and its three domain scores: Conceptual Skills (CON), Social Skills (SOC), and Practical Skills (PRA). The questionnaires were completed by the primary caregivers (98.5% of whom were mothers) concurrently with the WISC-IV assessments. To minimize subjective reporting bias, trained research assistants provided professional guidance to parents while they completed the ABAS-II, ensuring a standardized understanding of all assessment items.

#### 2.2.3. Competence–Performance Gap Index (CPGI)

To quantify the discrepancy between cognitive potential and adaptive performance, we calculated the Competence–Performance Gap Index (CPGI) using the standardized difference score method. The CPGI was defined as:

CPGI = Z(Cognitive_Mean) − Z(Adaptive_Mean).

where Z(Cognitive_Mean) represents the mean Z-score of the four WISC-IV indices (VCI, PRI, WMI, and PSI), and Z(Adaptive_Mean) represents the mean Z-score of the three ABAS-II domains (CON, SOC, and PRA). A CPGI > 0 indicates a cognitive advantage relative to adaptive performance (the “competence–performance gap”), whereas a CPGI < 0 indicates that adaptive performance exceeds measured cognitive potential. While conceptually similar to the “discrepancy score” (Hadjikhani et al., 2023) and “mismatch score” (Alvares et al., 2020) used in prior studies, our CPGI incorporates a broader range of cognitive dimensions instead of relying exclusively on the Full-Scale IQ (FSIQ).

### 2.3. Statistical Analysis

Latent Profile Analysis (LPA) was performed using Python 3.10 with the scikit-learn 1.6.1 library. We evaluated models ranging from one to five latent profiles. To determine the optimal solution, we assessed multiple fit indices—including AIC, BIC, sample-size adjusted BIC (aBIC), and Entropy for classification precision. We also used the Lo-Mendell-Rubin (LMR) and Bootstrap Likelihood Ratio Tests (BLRT) to confirm the statistical significance of adding additional profiles. In our LPA models, we assumed local independence and constrained variances to be equal across classes with covariances fixed to zero. Model selection was guided by a combination of statistical fit indices (AIC, BIC, and BLRT) and the theoretical interpretability of the identified profiles. Due to missing data on certain parental education variables, a listwise deletion approach was employed for the respective analyses. This resulted in slightly smaller sub-samples for models involving caregiver background, though the overall statistical power remained robust given the large initial sample size.

Following profile identification, one-way Analysis of Variance (ANOVA) was employed to examine inter-group differences, with Bonferroni correction applied for post-hoc multiple comparisons. To delineate the age-related patterns, linear regression models were used to fit the age-related trends of the CPGI within each profile. Furthermore, multinomial logistic regression was conducted to evaluate the predictive effects of sex, age, and parental education levels on profile membership, using the Global Delay group (Group 3) as the reference category. Statistical significance was defined as *p* < 0.05 (two-tailed).

## 3. Results

### 3.1. Latent Profile Analysis and Model Selection

As shown in Table 1, the information criteria (AIC, BIC, and aBIC) decreased consistently as the number of profiles increased. Balancing parsimony, clinical interpretability, and the distribution of subgroup sample sizes, the 3-profile model was ultimately selected as the optimal solution. This model demonstrated strong classification precision with an Entropy of 0.823, and both the LMR and BLRT results reached statistical significance (*p* < 0.001). The smallest profile accounted for 22.92% (n = 744) of the total sample, ensuring sufficient statistical power for subsequent comparative and regression analyses across all identified subgroups.

### 3.2. Participant Characteristics Across Latent Profiles

**Figure 1:**
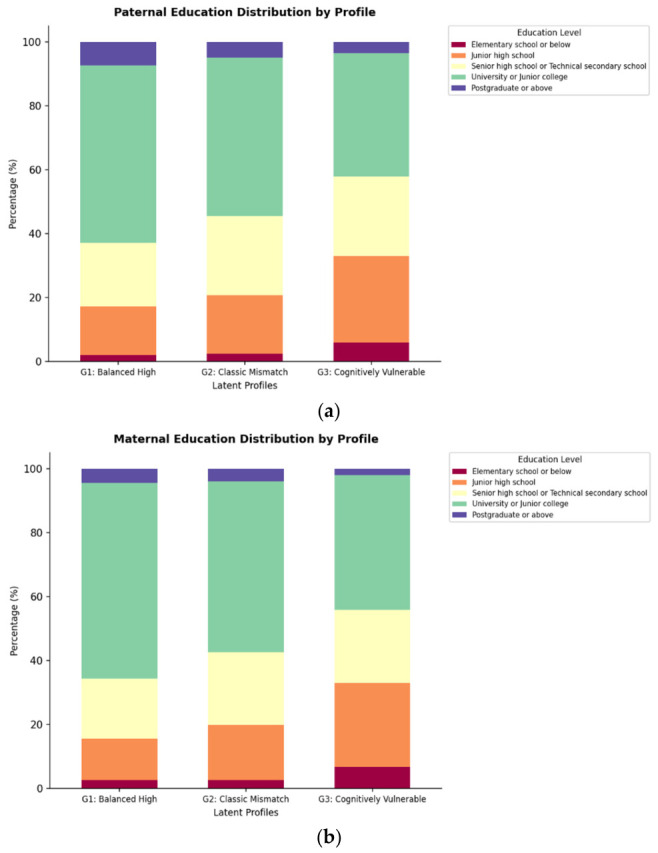
Distribution of Parental Education Across Profiles. Note: sub-figures (**a**) = Paternal Education Distribution by Profile; sub-figures (**b**) = Maternal Education Distribution by Profile.

Chi-square tests (Table 2) revealed significant differences in the distribution of sex across the three profiles (χ^2^ = 9.230, *p* = 0.010). Specifically, the proportion of female participants followed the descending order: Group 1 (25.7%) > Group 2 (24.5%) > Group 3 (19.8%). Furthermore, significant inter-group differences were observed in parental education levels (*p* < 0.001): Group 1 (the Balanced High-Functioning group) exhibited the highest proportion of parents with a college degree or above (55.6% for fathers and 61.3% for mothers). In contrast, Group 3 (the Cognitively Vulnerable group) had the largest concentration of parents with primary school education or below (5.9% for fathers and 6.7% for mothers) (Figure 1).

### 3.3. Characteristics and Comparisons of Cognitive and Adaptive Profiles

One-way ANOVA revealed significant differences across the three profiles for all WISC-IV indices (VCI, PRI, WMI, PSI, FSIQ) and ABAS-II domains (CON, SOC, PRA and GAC; all *p* < 0.001). Post-hoc comparisons further confirmed a significant gradient distribution for all assessed dimensions, following the order of Group 1 > Group 2 > Group 3 (Table 3). The specific functional characteristics of each profile are detailed below:

Group 1 (Balanced High, n = 1070): In this group, Z-scores for cognitive indices ranged between 0.3 and 0.5 (FSIQ = 98.52), while adaptive domain Z-scores approached or exceeded 1.0 (GAC = 106.88). This pattern indicates that their adaptive functioning slightly surpasses their measured cognitive potential, representing an optimal translation of ability into performance.

Group 2 (Classic Mismatch, n = 1432): This group is characterized by a relatively strong cognitive foundation, particularly in perceptual reasoning (PRI = 99.07), which is near the normative mean. However, their social adaptation significantly lags behind, with social (SOC = 87.79) and conceptual skills (CON = 88.25) falling into the lower range. This profile exemplifies a “high potential-low performance” dissociation model. To further evaluate clinical significance, we examined the prevalence of clinically meaningful discrepancies. In the overall sample (3246), 29.0% of children showed an IQ-GAC gap of ≥10 points, and 20.1% showed a gap of ≥15 points. Notably, in Group 2, while the mean difference was 4.77, 36.2% of children exceeded the 10-point threshold, and 24.8% showed a gap of ≥15 points. This suggested that the group-level trend is driven by a significant proportion of children with substantial functional impairments (see Supplementary Table S1). Specifically, these discrepancy rates refer to instances where FSIQ scores were higher than GAC scores, representing a ‘potential-performance gap.’ Differences in the opposite direction (GAC > FSIQ) account for a smaller percentage (6.6%) and are therefore not the focus of this analysis. A 15-point discrepancy (equivalent to 1.0 standard deviation) was adopted as the clinical threshold for a significant ‘cognitive–adaptive gap.’ This threshold was chosen because it significantly exceeds the standard error of difference (SEdiff) for the WISC-IV and ABAS-II, which typically ranges from 9 to 11 points at a 95% confidence level. By surpassing the SEdiff, this criterion ensures that the observed discrepancies are statistically reliable and unlikely to be the result of random measurement error or statistical chance.

**Figure 2:**
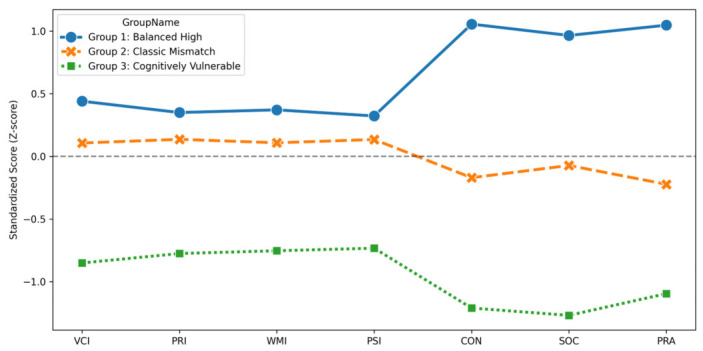
LPA Profile Plot. Note: The horizontal gray dashed line indicates the sample mean (Z = 0) as a reference point.

Group 3 (Cognitively Vulnerable, n = 744): Children in this group scored significantly below the mean across all cognitive and adaptive indices (Z-scores between −0.7 and −1.3). Both FSIQ (75.66) and GAC (71.84) were within the clinically impaired range, reflecting global deficits in both cognitive potential and adaptive functioning (Figure 2).

We also analyzed the differences in children’s FSIQ-GAC gaps across gender, grade level, and IQ level. These descriptive heterogeneous features provide a crucial empirical benchmark, showing that the small mean across the entire sample masks dramatic internal functional dissociations, further demonstrating the necessity of employing the individual-centered approach (LPA) to identify different functional profiles (see Supplementary Tables S1 and S2 for full details).

### 3.4. Age-Related Patterns of the Competence–Performance Gap Across Age

**Figure 3:**
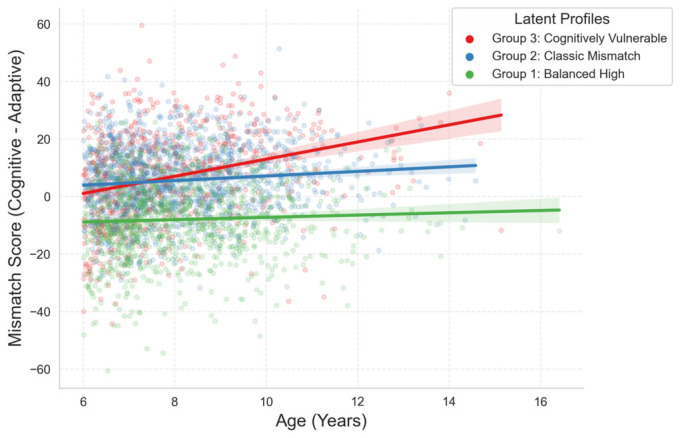
Scatter plot of the trend of age evolution and functional decoupling. Note: The three lines are fitted trend lines. The red line = Group 3 (Cognitively Vulnerable), the blue line = Group 2 (Classic Mismatch), and the green line = Group 1 (Balanced High).

Linear regression analysis revealed distinct phenotypic differences in the association between the Competence–Performance Gap Index (CPGI) and age (Figure 3). For Group 3 (Cognitively Vulnerable; red line), the CPGI exhibited a significant upward trend with age, suggesting that the gap between cognitive potential and adaptive performance progressively widens throughout the school-age period.

A similar upward trajectory was observed in Group 2 (Classic Mismatch; blue line); however, the slope was notably more gradual compared to Group 3. This indicates that while Group 2 also faces increasing functional challenges as they age, the rate of dissociation is significantly slower. In contrast, the regression line for Group 1 (Balanced High-Functioning; green line) remained at the lowest position on the scale and was nearly stationary across the age span. This stability demonstrates that children in Group 1 are able to consistently align their adaptive functioning with their cognitive potential during the school years, exhibiting superior developmental resilience.

### 3.5. Predictors of Profile Membership

As shown in Table 4, multinomial logistic regression analysis, using Group 3 (Cognitively Vulnerable) as the reference category, demonstrated that age, sex, and parental education levels were all significant predictors of functional profile membership in children with ASD. Regarding demographic factors, increasing age significantly raised the likelihood of being classified into Group 1 (OR = 1.178, *p* < 0.001) and Group 2 (OR = 1.166, *p* < 0.001). This suggests that compared to younger children, those in mid-to-late school age are more prone to exhibiting higher cognitive levels or the classic dissociation pattern rather than global functional impairment. Gender also significantly influenced developmental trajectories: girls were much more likely than boys to be classified into Group 1 (OR = 1.380, *p* = 0.007) and Group 2 (OR = 1.324, *p* = 0.014). This finding strongly supports the existence of a ‘female protective advantage’ in functional outcomes. In terms of the family environment, a significant asymmetry was observed in the predictive effects of paternal versus maternal education. Maternal education emerged as the strongest predictor of functional status, demonstrating a clear dose-response effect in preventing global vulnerability (Group 3). Specifically, compared to children of mothers with only a primary education, those whose mothers had bachelor’s or postgraduate degrees were 2.6 and 4.5 times more likely, respectively, to achieve the Group 2 functional profile (*p* < 0.001). A similar positive predictive effect was found for Group 1, where children of mothers with postgraduate degrees were 3.543 times more likely to achieve a balanced high-functioning profile (*p* = 0.004). In contrast, while paternal education demonstrated some predictive capacity, its impact was less extensive. Although highly educated fathers (Levels 4 and 5) significantly increased the odds of a child entering Group 1, their influence on Group 2 membership was only statistically significant at the senior high school (Level 3) and college (Level 4) levels. Overall, maternal educational background appears to exert a more central and robust influence on shaping the alignment between cognitive potential and adaptive functioning.

### 3.6. Hierarchical Regression on the Cognitive–Adaptive Discrepancy

To directly examine whether socio-environmental factors moderate the magnitude of the competence–performance gap, a hierarchical regression analysis was conducted with the FSIQ–GAC discrepancy (Gap) as the criterion variable (Table 5). In the first two steps, age and gender were entered as control variables. Age was found to be a significant positive predictor (*β* = 0.112, *p* < 0.001), suggesting that the discrepancy tends to increase with age. Gender did not significantly predict the gap magnitude (*p* = 0.114). In Step 3, parental education levels were added to the model. The results showed that maternal education significantly and negatively predicted the size of the gap (*B* = 1.316, *β* = 0.072, *p* = 0.003), whereas paternal education was not a significant predictor (*p* = 0.118). However, the addition of parental education only accounted for an incremental 1.0% of the variance (ΔR^2^ = 0.010, *p* < 0.001). While statistically significant, the minimal effect size indicates that parental education has a limited direct impact on the linear magnitude of the cognitive–adaptive discrepancy in children with ASD.

## 4. Discussion

By employing Latent Profile Analysis, the present study identified three distinct cognitive–adaptive phenotypes, unveiling significant phenotypic heterogeneity in the “competence–performance gap” among individuals with ASD. Our primary findings are threefold: First, the Classic Mismatch profile (44%) was the most prevalent group in our sample. This group follows a distinct dissociation model of high cognitive potential versus low adaptive performance. Such a finding suggests that for some of children with ASD(20.1% for the ≥15-point threshold), standardized IQ scores may overestimate functional capacity for a significant subset of children with ASD, particularly those within the Classic Mismatch group (44%). This highlights the importance of assessing both cognitive and adaptive domains to avoid a partial understanding of a child’s functional profile. When compared to normative data from the ABAS-II technical manual, which suggests that a 15-point FSIQ-GAC discrepancy occurs in only about 10–15% of the general population, the 20.1% prevalence observed in our ASD sample represents a significant elevation. This rate is even more pronounced in the Classic Mismatch group (24.8%), reinforcing the conclusion that this dissociation is a core functional characteristic for a substantial subset of children with ASD, rather than a result of statistical chance or measurement error. Second, the Cognitively Vulnerable profile (Group 3) exhibited a significant age-related widening of the competence–performance gap. This trend underscores a growing discrepancy between intensifying environmental demands and the relatively slow acquisition of functional skills as these children transition through school-age years. Third, our results highlight a statistically significant but weak association in the environmental moderation of the competence–performance gap, as maternal education exerted a more robust predictive influence on profile membership than paternal education. Collectively, these findings provide empirical evidence for understanding individual differences in the expression of intelligence and offer critical insights for optimizing clinical assessment and intervention practices for ASD.

In this study, Group 2 (Classic Mismatch) represented the largest subgroup (44.12%), characterized by a distinctive cognitive–adaptive dissociation. While their Perceptual Reasoning Index (PRI) fell within the average range, their social and conceptual skills significantly lagged behind. This “high-cognition–low-adaptation” pattern aligns with the theoretical framework of “discrepant intelligence,” which posits a substantial gap between nonverbal reasoning and daily functional adaptation (Alvares et al., 2020; Hadjikhani et al., 2023; McQuaid et al., 2021). Neuroimaging evidence suggests that this dissociation may stem from the asynchronous development of the “social brain” and executive function networks. Specifically, functional integration deficits in the temporoparietal junction (TPJ) and medial prefrontal cortex (mPFC) may impede the translation of abstract reasoning into real-world adaptive behaviors (Lau et al., 2026; S. Liu et al., 2025b; W. Song et al., 2025). Such children are often excluded from specialized intervention services due to their near-normal IQ scores, placing them in a clinical “gray zone” (Jolly & Barnard-Brak, 2024). This underscores the urgent need to establish screening criteria based on the “competence–performance gap” rather than relying solely on a single IQ threshold. In contrast, the Balanced High-Functioning group (Group 1) exhibited adaptive behaviors that slightly surpassed their cognitive performance, suggesting that a subset of children can achieve a relative cognitive–adaptive equilibrium. This subgroup likely possesses superior self-regulation and resource-integration capabilities, enabling them to fully leverage their relatively intact cognitive resources to learn and execute complex social-adaptive tasks (Nikolaos et al., 2023; Nuske et al., 2020). The success of these compensatory strategies in bridging the gap between ability and performance offers a crucial clinical insight: targeted adaptive skill training and contextualized teaching may facilitate functional independence even in children with borderline cognitive abilities. Finally, Group 3 (Cognitively Vulnerable) exhibited synchronized impairment in both cognitive and adaptive domains, supporting the “cognitive ceiling” effect. This effect suggests that when foundational cognitive processing capacities—specifically working memory and processing speed—fall below clinical thresholds, adaptive functioning becomes inherently constrained (Alexander & Reynolds, 2020). In such cases, isolated cognitive strategy training may be insufficient, necessitating more comprehensive, multi-modal supportive services. For children whose intelligence falls within the normal range, however, adaptive functioning appears to be more significantly modulated by post-natal environmental factors.

Through a dynamic analysis of the identified profiles, this study unveils a critical phenomenon in child development: the discrepancy between cognitive and adaptive functioning significantly intensifies with age, a trend most pronounced in the Cognitively Vulnerable group (Group 3). Our findings indicate that Group 3 exhibited the steepest upward slope in the CPGI. For these children, although cognitive abilities continue to develop, the rate of acquisition for social-adaptive skills fails to keep pace with the escalating environmental demands of the school system (O’Hearn & Lynn, 2022). As grade levels advance, the complexity of social interactions and the requirements for independent daily living increase exponentially. This leads to a widening chasm between cognitive potential and functional performance during mid-to-late childhood (Y. J. Chen et al., 2024; Sideropoulos et al., 2025). In contrast, while the Classic Mismatch group (Group 2) also showed signs of increasing dissociation over time, the progression was notably more gradual than that of Group 3. This suggests that children whose cognitive levels approach the normative mean may possess a “functional buffer” when navigating social demands (Livingston et al., 2019). Most striking is the Balanced High-Functioning group (Group 1), whose trajectory remained nearly stationary at a consistently low level. This indicates that these children not only maintain functional equilibrium throughout the school-age period but also effectively utilize social compensatory mechanisms to align, or even elevate, their adaptive performance relative to their cognitive baseline. The counterintuitive finding of accelerated dissociation in Group 3 may be explained by the mismatch between environmental press and developmental capacity (Gates et al., 2017). In this group, delayed cognitive development limits the foundation required for learning adaptive skills. Meanwhile, the exponential increase in societal expectations for independence and social nuance creates a dual-pressure environment, causing the functional gap to snowball. Unlike Group 2, which possesses the requisite cognitive scaffolding for skill acquisition, Group 3 faces a structural barrier. These diverging age-related trajectories suggest that intervention strategies must be proactive and predictive: for high-risk groups like Group 3, isolated cognitive training is insufficient to bridge the functional void. High-intensity social-adaptive and life-skills training must be prioritized in early childhood. Consequently, future intelligence research should transition from static score measurement to the longitudinal monitoring of intellectual translation pathways.

The diverging age-related patterns discussed above suggest a significant bifurcation in the functional prognosis of children with ASD. Family environmental resources—specifically parental educational background—likely shape this differentiation by influencing the accessibility and quality of early interventions. Our regression analysis demonstrates that both paternal and maternal education exert positive effects on a child’s functional development, a finding consistent with previous literature (Wilson, 2025). However, as maternal education levels rise, the likelihood of a child being classified into a more favorable functional profile (Group 1 or Group 2) shows a substantial, “stepped” increase. Notably, children of mothers with a postgraduate degree or higher are more than three times as likely to belong to Group 1 or Group 2 compared to those in the lowest education category. It is important to note that although maternal education was a significant predictor, it accounted for a very small proportion of the variance (0.4%). This suggests that while family environment is a relevant context, the “cognitive–adaptive gap” is primarily driven by internal neurodevelopmental factors inherent to ASD. This contrast suggests that the parental educational background exerts a nonlinear and probabilistic influence on functional outcomes in ASD, rather than a simple additive one. Specifically, the logistic model suggests a “threshold effect” for environmental scaffolding. High maternal education—particularly at the postgraduate level—acts as a catalyst, significantly increasing the likelihood that a child moves from a “Cognitively Vulnerable” profile toward more cognitively capable groups. This suggests that highly educated mothers often possess sharper developmental observation skills, superior resource-acquisition capabilities, and higher adherence to intervention protocols. This also indicated that the cognitive–adaptive gap in children with ASD is primarily driven by intrinsic neurodevelopmental factors, with environmental factors (such as maternal education) playing only a very minor moderating role. The significance of maternal education may more accurately reflect the reporting style of highly educated mothers in assessments or provide weak compensatory support to the child. Collectively, these factors construct a robust risk-protection barrier, effectively preventing a child’s functioning from sliding into the most severe state of global impairment (Al-Hamza Marhoon & Obaid, 2025; Casula et al., 2024; W. Li et al., 2024). This finding implies that high-quality family resources may be constrained by a child’s neurobiological foundation, limiting the absolute “ceiling” of functioning. Nevertheless, they significantly elevate the functional “floor” or baseline for survival and adaptation. In contrast, while paternal education also positively predicts membership in the high-functioning group which was consistent with prior research (Shahid Khan et al., 2024), its breadth and statistical significance are notably less pervasive than those of maternal education. This disparity underscores that within the early rearing and intervention ecosystem, mothers typically assume a more direct and central role in resource translation (Kreiser & Segal, 2025). While both factors contribute to the family environment, maternal education emerged as a more robust predictor, which could be found in previous studies (Dong et al., 2022; Kim et al., 2026; Lindsay Olson et al., 2021). This could be attributed to several mechanisms. First, in the current cultural context, mothers often serve as the primary caregivers, and their educational background directly translates into the quality of daily cognitive stimulation and adaptive skill-modeling. Second, high maternal education is frequently associated with increased engagement in evidence-based intervention programs, which specifically target the closure of the functional gap. Finally, cultural expectations often place a higher emphasis on the mother’s role in managing the child’s social and adaptive development, making their influence more direct compared to paternal influence (Doyumgac et al., 2026). Our results argue that environmental factors are not merely a passive backdrop for cognitive development. Instead, they serve as a catalyst for converting intelligence from a “potential state” to a “functional state,” highlighting its socially constructed nature. Meantime, the robust association between parental education and child functional profiles likely involves several overlapping mechanisms. First, parental education often serves as a proxy for parental cognitive ability, suggesting that the link between higher maternal education and the “Balanced High-Functioning” profile may partially reflect the heritability of cognitive potential (Dardani et al., 2022; X. Liu et al., 2025). Second, from an environmental perspective, higher education and SES provide families with the socioeconomic capital necessary to foster “environmental scaffolding”. Highly educated parents may be better equipped to provide structured opportunities for independence and to navigate complex healthcare systems to advocate for evidence-based interventions (Burke et al., 2023).

The present study reveals that sex plays a pivotal role in predicting functional profile membership. Multinomial logistic regression analysis indicated that, compared to boys, girls had a 1.380-fold higher likelihood of belonging to the Balanced High-Functioning group (Group 1) and a 1.324-fold higher likelihood of being classified into the Classic Mismatch group (Group 2), relative to the Cognitively Vulnerable group (Group 3). These findings suggest that given similar neurodevelopmental backgrounds, girls are less prone than boys to slide into the severe, global impairment characterized by Group 3, indicating a potential functional prognostic advantage for females. This advantage may stem from innate biological resilience or a stronger drive for social imitation (Simcoe et al., 2023). During the school-age years, many girls with ASD are able to “mask” their social deficits by observing and mimicking the behaviors of their neurotypical peers (Milner et al., 2023; Ochoa-Lubinoff et al., 2023). This compensatory mechanism allows them to achieve higher scores on adaptive behavior assessments, resulting in their over-representation in Groups 1 and 2. However, this protective advantage is a “double-edged sword.” While it makes their functional performance appear closer to that of typically developing children, it significantly elevates the risk of misdiagnosis or late diagnosis (Lockwood Estrin et al., 2021; Saure et al., 2023). Because their social challenges are often concealed beneath relatively intact cognitive abilities and sophisticated social camouflaging behaviors, they do not exhibit the global impairments seen in Group 3. Consequently, their underlying difficulties are more likely to be overlooked by parents and educators, delaying access to necessary support services (Gindi et al., 2026).

The present study has several limitations that warrant consideration. First, due to the cross-sectional nature of the design, the observed trends in cognitive–adaptive dissociation reflect inter-group differences rather than intra-individual developmental changes. Future longitudinal studies are essential to validate these “emergent deficits” as children age. Second, the assessment of adaptive functioning relied on caregiver reports, which may be subject to informant bias or parental expectations. Integrating multi-informant assessments and direct clinical observations would yield a more objective profile. Finally, this study did not account for critical confounding variables such as psychiatric comorbidities (e.g., ADHD) or intervention history. Moreover, maternal education serves as only one facet of the family ecosystem; future research should employ a broader social-ecological framework to explore the comprehensive impact of family dynamics and social support systems on the functional prognosis of school-aged children with ASD. Another important consideration is the potential confounding effect of therapeutic interventions. While our results indicate a significant gap between cognitive potential and adaptive mastery, it is possible that the intensity, duration, and type of rehabilitation programs (e.g., Applied Behavior Analysis or social skills training) received by the participants may have modulated these outcomes. Due to the cross-sectional nature of this clinical sample, we were unable to systematically control for high-resolution intervention data, such as weekly treatment hours or specific pedagogical approaches. Future longitudinal studies should incorporate detailed intervention histories to better isolate the independent contribution of family environmental factors from the effects of professional therapeutic support. Finally, the findings regarding the role of parental education must be interpreted with caution due to potential “informant bias”. Specifically, more highly educated parents may possess more nuanced or stringent developmental expectations, which could lead to a more critical appraisal of their child’s adaptive performance on the ABAS-II. While we attempted to mitigate this by providing professional guidance during the assessment process, the subjective nature of parental reports remains a limitation. The observed cognitive–adaptive gap may, in part, reflect a complex interaction between the child’s actual functional skills and the lens through which parents observe and report those skills.

## 5. Conclusions

By employing Latent Profile Analysis (LPA), the present study identifies distinct heterogeneous profiles of the cognitive–adaptive relationship in school-aged children with ASD. Our results confirm that the “competence–performance gap” is not a stochastic deviation but a constitutive intellectual phenotype, particularly prominent in children with global functional impairments. From a dynamic developmental perspective, children with severe functional deficits face an accelerating risk of dissociation as they age, suggesting a potential attenuation in the efficiency of intellectual translation during critical developmental windows. The paramount theoretical contribution of this research lies in elucidating the role of maternal education as a pivotal family ecological factor. This “scaffolding” association is crucial for bridging the gap between cognitive potential and adaptive behaviors. It creates a protective barrier for long-term functional outcomes, highlighting how environmental support fundamentally shapes real-world intelligence. Our findings suggest a subtle but significant association between maternal education and cognitive–adaptive profiles, particularly in promoting balanced development during the school-age years. However, the small proportion of variance explained indicates that other unmeasured factors (e.g., specific interventions, biological factors) likely play a more dominant role. Consequently, we advocate that clinical assessments incorporate the degree of dissociation as a standardized metric for ASD functional evaluation. Furthermore, by integrating family resources, clinicians should provide personalized, lifespan-oriented adaptive support tailored to different cognitive profiles. Such an approach is essential to maximize the social value of intellectual potential and improve the quality of life for children with ASD across the school-age trajectory.

## Figures and Tables

**Table 1:** LPA Model comparison.

Models	AIC	BIC	aBIC	Entropy	LMR (p)	BLRT (p)	Min Class
1-Profile	64,491.33	64,576.52	64,532.04	-	-	-	100%
2-Profile	58,720.76	58,897.23	58,805.08	0.823	<0.001	<0.001	40.40%
**3-Profile**	**56,336.54**	**56,604.29**	**56,464.48**	**0.823**	<**0.001**	<**0.001**	**22.92%**
4-Profile	54,919.93	55,278.96	55,091.49	0.837	<0.001	<0.001	8.70%
5-Profile	53,849.69	54,299.99	54,064.86	0.820	<0.001	<0.001	7.00%

Note: The bolded line represents the optimal solution ultimately selected in this study.

**Table 2:** Participant Characteristics.

Variables	Total	Group 1 Balanced High	Group 2 Classic Mismatch	Group 3 Cognitively Vulnerable	F/χ^2^	*p*	*η_p_* ^2^
Gender n (%)	3246	1070	1432	744	9.230	0.010	-
Boy	2473 (76.2)	795 (74.3)	1081 (75.5)	597 (80.2)			
Girl	773 (23.8)	275 (25.7)	351 (24.5)	147 (19.8)			
Age M (SD)	8.034 (1.054)	8.087 (1.484)	8.096 (1.482)	7.841 (1.559)	7.906	<0.001	0.005
Paternal Education n (%)	3229	1076	1423	730	103.227	<0.001	-
Level 1	99 (3.1)	21 (2.0)	35 (2.5)	43 (5.9)			
Level 2	624 (19.3)	164 (15.2)	262 (18.4)	198 (27.1)			
Level 3	745 (23.1)	214 (19.9)	350 (24.6)	181 (24.8)			
Level 4	1586 (49.1)	598 (55.6)	706 (49.6)	282 (38.6)			
Level 5	175 (5.4)	79 (7.3)	70 (4.9)	26 (3.6)			
Maternal Education n (%)	3220	1068	1422	730	114.391	<0.001	-
Level 1	113 (3.5)	27 (2.5)	37 (2.6)	49 (6.7)			
Level 2	577 (17.9)	138 (12.9)	247 (17.4)	192 (26.3)			
Level 3	691 (21.5)	201 (18.8)	323 (22.7)	167 (22.9)			
Level 4	1721 (53.4)	655 (61.3)	758 (53.3)	308 (42.2)			
Level 5	118 (3.7)	47 (4.4)	57 (4.0)	14 (1.9)			

Level 1 = Elementary school or below; Level 2 = Junior high school; Level 3 = Senior high school or Technical secondary school; Level 4 = University or Junior college; Level 5 = Postgraduate or above.

**Table 3:** Comparison of Cognitive and Adaptive Indices Among the Three Latent Profiles.

Variables	Group 1	Group 2	Group 3	F	*p*	*η_p_* ^2^	Post Hoc
WISC-IV							
VCI	100.35 (15.975)	94.33 (14.571)	77.18 (17.464)	492.891	<0.001	0.233	1 > 2 > 3
PRI	102.77 (15.372)	99.07 (14.276)	83.22 (18.708)	366.610	<0.001	0.184	1 > 2 > 3
WMI	93.63 (13.949)	89.54 (12.657)	76.41 (15.870)	354.666	<0.001	0.179	1 > 2 > 3
PSI	96.19 (13.751)	93.16 (13.100)	79.50 (17.610)	318.500	<0.001	0.164	1 > 2 > 3
FSIQ	98.52 (14.085)	92.96 (12.375)	75.66 (15.781)	631.154	<0.001	0.280	1 > 2 > 3
ABAS-II							1 > 2 > 3
CON	106.22 (9.184)	88.25 (6.822)	73.02 (7.633)	4035.771	<0.001	0.713	1 > 2 > 3
SOC	104.72 (10.016)	87.79 (8.312)	68.27 (10.025)	3376.491	<0.001	0.676	1 > 2 > 3
PRA	107.77 (9.568)	89.50 (6.888)	76.93 (9.428)	3096.798	<0.001	0.656	1 > 2 > 3
GAC	106.88 (8.329)	88.19 (6.057)	71.84 (8.340)	5027.433	<0.001	0.756	1 > 2 > 3

Notes: VCI = Verbal Comprehension Index; PRI = Perceptual Reasoning Index; WMI = Working Memory Index; PSI = Processing Speed Index; FSIQ = Full-scale Intelligence Quotient; CON = Conceptual Composite; SOC = Social Composite; PRA = Practical Composite; GAC = General Adaptive Composite.

**Table 4:** Multinomial logistic regression for predicting subgroup affiliation.

Predictors	Comparison Group	*β*	SE	OR	*p*	95% CI
Intercept	G1 vs. G3	−2.399	0.435	-	<0.001	-
	G2 vs. G3	−1.927	0.397	-	<0.001	-
Age	G1 vs. G3	0.164	0.035	1.178	<0.001	[1.101,1.261]
	G2 vs. G3	0.154	0.033	1.166	<0.001	[1.093,1.244]
Gender	G1 vs. G3	0.322	0.120	1.380	0.007	[1.092,1.745]
	G2 vs. G3	0.281	0.114	1.324	0.014	[1.059,1.655]
Maternal Education						
Level 2	G1 vs. G3	0.181	0.281	1.198	0.520	[0.691,2.079]
	G2 vs. G3	0.469	0.253	1.598	0.064	[0.972,2.625]
Level 3	G1 vs. G3	0.602	0.289	1.826	0.037	[1.036,3.216]
	G2 vs. G3	0.810	0.262	2.248	0.002	[1.346,3.758]
Level 4	G1 vs. G3	0.972	0.295	2.643	0.001	[1.483,4.711]
	G2 vs. G3	0.969	0.269	2.635	<0.001	[1.556,4.464]
Level 5	G1 vs. G3	1.265	0.445	3.543	0.004	[1.481,8.482]
	G2 vs. G3	1.502	0.423	4.491	<0.001	[1.958,10.288]
Paternal Education						
Level 2	G1 vs. G3	0.447	0.305	1.564	0.143	[0.860, 2.841]
	G2 vs. G3	0.341	0.263	1.406	0.194	[0.840, 2.356]
Level 3	G1 vs. G3	0.568	0.312	1.765	0.069	[0.957, 3.254]
	G2 vs. G3	0.558	0.270	1.747	0.039	[1.028, 2.965]
Level 4	G1 vs. G3	0.879	0.319	2.408	0.006	[1.289, 4.493]
	G2 vs. G3	0.638	0.279	1.893	0.022	[1.096, 3.271]
Level 5	G1 vs. G3	1.076	0.403	2.933	0.008	[1.331, 6.456]
	G2 vs. G3	0.500	0.375	1.649	0.182	[0.791, 3.438]

Note: Reference categories for categorical predictors: “Elementary school or below” for parental education levels (Maternal and Paternal Education), and “Boy” for gender. The reference profile for the multinomial logistic regression is Group 3 (Cognitively Vulnerable).

**Table 5:** Hierarchical Regression Analysis Predicting the Cognitive–Adaptive Discrepancy (Gap).

Predictors	*B*	SE	*β*	t	*p*	R^2^	ΔR^2^
Step 1						0.012	-
Age	1.250	0.199	0.110	6.292	<0.001		
Step 2						0.013	0.001
Age	1.263	0.199	0.112	6.354	<0.001		
Gender	−1.107	0.701	−0.028	−1.580	0.114		
Step 3						0.023	0.010 **
Age	1.386	0.199	0.122	6.969	<0.001		
Gender	−1.237	0.698	−0.031	−1.773	0.076		
Maternal Education	1.316	0.449	0.072	2.932	0.003		
Paternal Education	0.688	0.440	0.038	1.565	0.118		

Note: **: *p* < 0.001; The criterion variable is the FSIQ–GAC discrepancy (Gap). Gender was coded as Male = 1, Female = 2. ΔR^2^ for Step 3 is based on the addition of both paternal and maternal education.

## Data Availability

The data presented in this study are available on request from the corresponding author due to privacy.

## Supplementary Materials

The following supporting information (Supplementary Tables S1 and S2) can be downloaded at: https://www.mdpi.com/article/10.3390/jintelligence14060103/s1.

## Abbreviations

The following abbreviations are used in this manuscript:

ASD	Autism spectrum disorder
IQ	Intelligence Quotient
LPA	Latent Profile Analysis
SES	Socioeconomic Status
WISC-IV	Wechsler Intelligence Scale for Children, Fourth Edition
ABAS-II	Adaptive Behavior Assessment System, Second Edition
CPGI	Competence–Performance Gap Index
FSIQ	Full-Scale Intelligence Quotient
VCI	Verbal Comprehension Index
PRI	Perceptual Reasoning Index
WMI	Working Memory Index
PSI	Processing Speed Index
GAC	General Adaptive Composite
CON	Conceptual Skills
SOC	Social Skills
PRA	Practical Skills
AIC	Akaike Information Criterion
BIC	Bayesian Information Criterion
aBIC	adjusted BIC
LMR	Lo-Mendell-Rubin
BLRT	Bootstrap Likelihood Ratio Test
