# Supplementary material for: Individual Differences in the “Cognitive–Adaptive Gap” Among Children with Autism Spectrum Disorder: A Latent Profile Analysis of the Moderating Role of Family Environment
Source: J Intell. 2026 Jun 9;14(6):103. doi: 10.3390/jintelligence14060103 (PMC13300842; doi:10.3390/jintelligence14060103)
Supplement: Supplementary file 1 [file jintelligence-14-00103-s001.zip › jintelligence-4198426-supplementary.pdf]

Supplementary Table S1. Prevalence of Clinically Meaningful IQ-GAC Discrepancies Across Latent Profiles

Profile	Mean Gap (SD)	Gap≥10 points (%)	Gap≥15 points (%)
Total Sample (n=3246)	0.19 (17.05)	29.0	20.1
Group 1 (n=1070) Balanced High	-8.36 (16.28)	13.5	8.8
Group 2 (n=1432) Classic Mismatch	4.77 (14.43)	36.2	24.8
Group 3 (n=744) Cognitively Vulnerable	3.82 (18.11)	37.5	27.3

Note: IQ-GAC gap was calculated as Full-Scale IQ (WISC-IV) minus General Adaptive Composite (ABAS-II). A discrepancy of ≥15 points corresponds to one standard deviation, a common threshold for clinical significance.

Supplementary Table S2. Distribution of FSIQ–GAC Discrepancy Scores Across Subgroups

	N	Mean Gap (SD)	t/F	*p*
Sex			2.578	0.176
Boys	2473	0.42(17.19)		
Girls	773	-0.54(16.57)		
Grade			29.957	< 0.001
Kindergarten or below	343	-6.94(17.08)		
Grade 1~2	1838	-0.05(16.76)		
Grade 3~4	848	3.06(16.80)		
Grade≥5	217	2.20(17.00)		
FSIQ			541.430	< 0.001
≤70	270	-17.17(12.47)		
70-90	1240	-6.56(14.42)		
≥90	1736	7.71(15.33)		
